# Synthesis, Crystal Structures, and Spectroscopic Characterization of Bis-aldehyde Monomers and Their Electrically Conductive Pristine Polyazomethines

**DOI:** 10.3390/polym11091498

**Published:** 2019-09-13

**Authors:** Abdul Hafeez, Zareen Akhter, John F. Gallagher, Nawazish Ali Khan, Asghari Gul, Faiz Ullah Shah

**Affiliations:** 1 Department of Chemistry, Quaid-i-Azam University, Islamabad 45320, Pakistan; 2 School of Chemical Sciences, Dublin City University, Glasnevin, Dublin 9, Ireland; 3 Materials Science Laboratory, Department of Physics, Quaid-i-Azam University, Islamabad 45320, Pakistan; 4 Department of Chemistry, COMSATS University, Islamabad 45320, Pakistan; 5 Chemistry of Interfaces, Luleå University of Technology, 971 87 Luleå, Sweden

**Keywords:** Polyazomethines, single crystals, bis-aldehydes, spectroscopic characterization, conductivity, poly(Schiff base)s

## Abstract

Bis-aldehyde monomers 4-(4′-formyl-phenoxy)benzaldehyde (**3a**), 3-methoxy-4-(4′-formyl-phenoxy)benzaldehyde (**3b**), and 3-ethoxy-4-(4′-formyl-phenoxy)benzaldehyde (**3c**) were synthesized by etherification of 4-fluorobenzaldehyde (**1**) with 4-hydroxybenzaldehyde (**2a**), 3-methoxy-4-hydroxybenzaldehyde (**2b**), and 3-ethoxy-4-hydroxybenzaldehyde (**2c**), respectively. Each monomer was polymerized with *p*-phenylenediamine and 4,4′-diaminodiphenyl ether to yield six poly(azomethine)s. Single crystal X-ray diffraction structures of **3b** and **3c** were determined. The structural characterization of the monomers and poly(azomethine)s was performed by FT-IR and NMR spectroscopic techniques and elemental analysis. Physicochemical properties of polymers were investigated by powder X-ray diffraction, thermogravimetric analysis (TGA), viscometry, UV–vis, spectroscopy and photoluminescence. These polymers were subjected to electrical conductivity measurements by the four-probe method, and their conductivities were found to be in the range 4.0 × 10^−5^ to 6.4 × 10^−5^ Scm^−1^, which was significantly higher than the values reported so far.

## 1. Introduction

Conjugated organic semiconducting polymers have emerged as new materials having potential applications in electronics, opto-electronics, and photonics [1,2,3]. Polyazomethines, also known as poly(Schiff base)s or polyimines, with general structure (R–CH=N–R′), are a group of organic conducting polymers [4,5] exhibiting excellent thermal [6,7,8], and mechanical properties. Conjugated polyazomethines show interesting optical [9,10,11,12], optoelectronic [13,14,15], and electrical [6,16,17,18] properties.

The imine bond (–CH=N–) is isoelectronic with the vinylene bond (–CH=CH–) [4,5,11,19], and the replacement of R and R′ with aromatic groups results in the formation of a conjugated structure. When this conjugation extends throughout the polymer, this forms the basis of electronic conduction. Apart from that, their synthetic procedures, which are carried out under mild conditions, require condensation polymerization of dialdehydes with diamines, in a very simple way. This is unlike Suzuki [20], Wittig [21], Stille [22], or Heck carbon–carbon coupling reactions [23,24], commonly carried out for polymerization of carbon–carbon-linked conjugated structures [25]. Products obtained from those coupling reactions, in addition, require a cumbersome purification process and isolation of catalysts [26,27].

On the other hand, synthesis of polyazomethines might be catalyzed with mineral or organic acids, and the product is purified by a simple precipitation method. The conjugated polyazomethines are widely used as a counterpart for currently employed conjugated structures in optoelectronic devices [28] such as photovoltaic cells [29,30] and electroluminescent [19] and electrochromic [1,16,31] devices. Still, there are some practical problems with their low conductivity.

As for other many conjugated polymers, various properties of polyazomethines, including conductivity, can be improved by suitable doping or protonating processes [32]. Over the past few decades, halogens (such as Br, Cl, I) have been the most commonly used doping elements. The effects of protonating and doping on the properties of imine (–CH=N–) polymers have been investigated in numerous papers [8,16,33,34,35]. The mechanism of iodine oxidative doping has been reported in previous publications [3,36], as the process of removing an electron from the lone pair of nitrogen atom. However, according to our best knowledge, the conductivity of solely conjugated polyazomethines has not been reported yet. Those which have been reported contain different nonconjugated aliphatic spacers [6,34].

Therefore, the conductivity of wholly aromatic pristine polyazomethines is being reported by us for the first time. Herein, we report the syntheses of three bis-aldehyde monomers and their polymers. The spectroscopic characterizations accompanied by single crystal X-ray diffraction profiles are performed. The electrical conductivity is measured by using pelletized polymer samples. The expected structure–property relationship has been explained.

## 2. Experimental Section

### 2.1. Materials

The chemicals K_2_CO_3_ anhydrous (Sigma Aldrich, Hamburg, Germany), 4,4′-diaminodiphenyl ether (97%, melting point = 188–192 °C, Sigma Aldrich), 1,4-phenylenediamine (melting point = 138–143 °C, Sigma Aldrich), and *p*-toluene sulfonic acid monohydrate (PTSA) Fluka (Buchs, Switzerland) were purchased from commercial suppliers. 4-Fluorobenzaldehyde (98%), 4-hydroxybenzaldehyde (97%, melting point = 284–285 °C), 3-methoxy-4-hydroxybenzaldehyde (m.p = 158–159 °C), and 3-ethoxy-4-hydroxybenzaldehyde (melting point = 160–163 °C) were obtained from Sigma Aldrich. The solvents *n*-hexane, dimethylsulfoxide, and diethyl ether were purchased from Merck (Darmstadt, Germany), whereas ethylacetate, chloroform, and toluene were obtained from Panreac (Barcelona, Spain). The methanol, ethanol, and *N*,*N*-dimethylformamide were purchased from Sigma Aldrich, whereas hydrochloric acid and sulfuric acid were obtained from Riedel-de-Haen (Hannover, Germany).

### 2.2. Physical Characterizations

The melting points of the synthesized aromatic bis-aldehyde monomers were determined using a Mel-Temp. (Mitamura Riken Rogyo, Inc. Tokyo, Japan) apparatus with open capillary tubes. Fourier transform infrared (FTIR) spectroscopy was performed using a Perkin Elmer 1600 series FTIR spectrophotometer (Waltham, MA, USA). Nuclear magnetic resonance (NMR) measurements were carried out using Bruker Avance 300 digital NMR (Billerica, MA, USA) in different solvents depending on the solubility of the samples. Tetramethylsilane was used as an internal standard. Elemental analyses were performed on a Vaio-EL instrument. The UV–visible spectra of azomethine polymers were measured on Schimadzu-1700 UV using chloroform (CHCl_3_), dimethylformamide (DMF), and sulfuric acid. Thermogravimetric analyses (TGAs) were performed on a Perkin Elmer TGA instrument v.7 at heating rates of 10 and 20 °C/min in nitrogen or air up to a maximum 650–950 °C. Photoluminescence spectra of the polyazomethines were measured on a Perkin Elmer LS 55 luminescence instrument using a single glass cell. The viscosities of polyazomethines were measured at room temperature using a U-tube Ubbelhode viscometer with 20 mL of the polymer solutions. Wide-angle X-ray diffraction experiments of the powdered polymers samples were performed using Philips 3040/60 X′Pert Pro diffractometer having a Cu anode with a Kα radiation source. The electrical conductivity (*σ*) of polyazomethines was measured with a Keithley source meter 2400. The crystal structure data of the monomers were collected using an Oxford diffraction diffractometer (Mo radiation), structures using CrysAlisPro, and were solved and refined using the SHELXS/SHELXL14 programs and diagrams prepared by using the Mercury program. The crystal structure analysis was performed using the PLATON package and the OSCAIL suite of programs [37,38,39,40,41].

### 2.3. Syntheses of Monomers (**3a** to **3c**)

**Figure 1:**
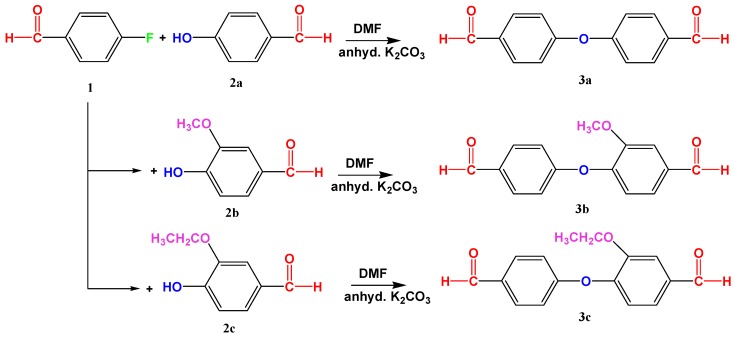
Synthesis of aromatic bis-aldehyde monomers by reacting 4-fluorobenzaldehyde (**1**) with 4-hydroxybenzaldehyde (**2a**), 3-methoxy-4-hydroxygenzaldehyde (**2b**), and 3-ethoxy-4-hydroxybenzaldehyde (**2c**). The synthesized monomers are 4-(4′-Formyl-phenoxy)benzaldehyde (**3a**), 3-Methoxy-4-(4′-formyl-phenoxy)benzaldehyde (**3b**), and 3-Ethoxy-4-(4′-formyl-phenoxy)benzaldehyde (**3c**).

The syntheses of three monomers, **3a-c**, is depicted in Figure 1. In a typical experiment a magnetic stirrer, 0.2 mmol of **2a-c**, and 0.2 mmol of anhydrous K_2_CO_3_ were taken in 100 mL of two neck round-bottom flask along with 15 mL of dried DMF. The contents of the flask were gently heated with a slow temperature ramping to 80 °C for 1 h. Afterwards, the flask was allowed to cool to ambient temperature before the addition of 0.2 mmol of 4-fluorobenzaldehyde (**1**), and stirring continued for a further 1 h. The reaction mixture was heated to reflux under a nitrogen atmosphere at 120 °C for 24 h. When the reaction completed, as indicated by TLC, the reaction mixture was poured into 200 mL of ice-cooled water, and the base was neutralized with 10% HCl solution. The precipitates were allowed to stand for 40 min, then filtered with filter paper through gentle suction. Later on, the products were dried in the oven at 40 °C before recrystallization with CHCl_3_/ethylacetate. After recrystallization, the precipitates were isolated for analysis and further polymerization.

#### 4-(4′-.Formyl-phenoxy)benzaldehyde (**3a**)

The same experimental conditions were used as described above for coupling of 4-fluorobenzaldehyde (**1**) and 4-hydroxybenzaldehyde (**2a**) to form crystalline solid **3a**, yield 90%, m.p 55–60 °C. IR (KBr): *υ*(Ar–H str.), 3065 cm^−1^; *υ*(aldehydic C–H str.), 2819, 2731 cm^−1^; *υ*(C=O str.), 1675 cm^−1^; *υ*(aromatic C=C), 1581, 1494 cm^−1^; *υ*(C–O–C ether), 1236 cm^−1^. ^1^H NMR (300 MHz, CDCl_3_) *δ* 9.99 (s, 2H), 7.94 (d, *J* = 8.0 Hz, 4H), 7.19 (d, *J* = 8.0 Hz, 4H). ^13^C NMR (75 MHz, CDCl_3_) *δ* 190.64 (s), 161.01 (s), 132.34 (d, *J* = 38.2 Hz), 119.37 (s). Elem. Anal. Calc.: C_14_H_10_O_3_: C, 74.33; H, 4.46; O, 21.22. Found: C, 75.08; H, 4.93%.

#### 3-Met.hoxy-4-(4′-formyl-phenoxy)benzaldehyde (**3b**)

The bis-aldehyde **3b** was synthesized by the protocol as described for **3a** using 3-methoxy-4-hydroxygenzaldehyde (**2b**) and 4-fluorobenzaldehyde (**1**) to yield crystalline solid with 88% yield, and m.p 67–68 °C. IR (KBr): *υ*(Ar–H str.), 3066 cm^−1^; *υ*(aliphatic C–H str.), 2978, 2928 cm^−1^; *υ*(aldehydic C–H str.), 2860, 2759 cm^−1^; *υ*(C=O str.), 1680 cm^−1^; *υ*(aromatic C=C), 1581, 1497 cm^−1^; *υ*(C–O–C ether), 1235, 1208 cm^−1^. ^1^H NMR (300 MHz, CDCl_3_) *δ* 9.98 (s, 1H), 9.95 (s, 1H), 7.88 (d, *J* = 8.6 Hz, 2H), 7.58 (s, 1H), 7.52 (d, *J* = 9.7 Hz, 1H), 7.20 (d, *J* = 8.0 Hz, 1H), 7.07 (d, *J* = 8.6 Hz, 2H), 3.90 (s, 3H). ^13^C NMR (75 MHz, CDCl_3_) *δ* 190.74 (s), 162.02 (s), 152.00 (s), 148.95 (s), 134.15 (s), 131.96 (s), 125.45 (s), 121.40 (s), 117.41 (s), 111.25 (s), 56.11 (s). Elem. Anal. Calc.: C_15_H_12_O_4_: C, 70.31; H, 4.72; O, 24.97. Found: C, 71.05; H, 4.24%.

#### X-ray Crystallographic Data of **3b**

**Figure 2:**
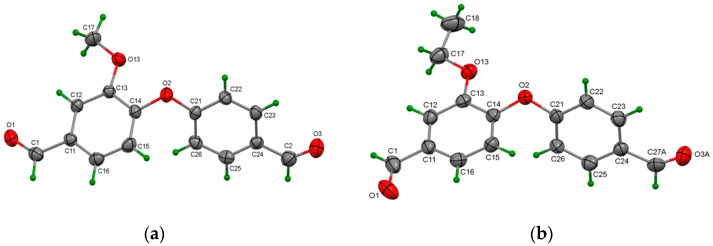
(**a**) ORTEP diagram of monomer **3b**, and (**b**) ORTEP diagram of monomer **3c**.

C_15_H_12_O_4_, M = 256.25, orthorhombic, space group *Pbcn*, *a* = 18.4038(11), *b* = 8.6240(5), *c* = 15.7283(11) Å, V = 2496.3(3) Å^3^, *Z* = 8, *Z*′ = 1, *R*-factor (%) 5.30, 12.5. The Oak Ridge Thermal Ellipsoid Plot (ORTEP) diagram of **3b** is depicted in Figure 2a.

#### 3-Eth.oxy-4-(4′-formyl-phenoxy)benzaldehyde (**3c**)

The **3c** monomer was also synthesized by the above protocol using 3-ethoxy-4-hydroxybenzaldehyde (**2c**) and 4-fluorobenzaldehyde (**1**) to yield crystalline solid with 85% yield and m.p 68–70 °C. IR (KBr): *υ*(Ar–H str.), 3063 cm^−1^; *υ*(aliphatic C–H str.), 2973, 2930 cm^−1^; υ(aldehydic C–H str.), 2828, 2736 cm^−1^; *υ*(C=O str.), 1687 cm^−1^; *υ*(aromatic C=C), 1577, 1497 cm^−1^; υ(C–O–C ether), 1268, 1227 cm^−1^. ^1^H NMR (300 MHz, CDCl_3_) *δ* 9.96 (s, 1H), 9.95 (s, 1H), 7.87 (d, *J* = 8.6 Hz, 2H), 7.54 (s, 1H), 7.50 (d, *J* = 9.4 Hz, 1H), 7.22 (d, *J* = 8.0 Hz, 1H), 7.06 (d, *J* = 8.6 Hz, 2H), 4.11 (q, *J* = 6.9 Hz, 2H), 1.27 (t, *J* = 7.0 Hz, 3H). ^13^C NMR (75 MHz, CDCl_3_) δ 190.90 (s), 190.76 (s), 162.37 (s), 151.31 (s), 149.08 (s), 134.20 (s), 131.82 (s), 125.19 (s), 121.79 (s), 117.28 (s), 112.40 (s), 64.64 (s), 14.40 (s). Elem. Anal. Calc.: C_16_H_14_O_4_: C, 71.10; H, 5.22; O, 23.68. Found: C, 70.23; H, 4.37%.

#### X-ray Crystallographic Data of **3c**

C_16_H_16_O_4_, *M_w_* = 270.27, monoclinic, space group *P*2_1_/c, *a* = 8.8926(7), *b* = 19.2717(11), *c* = 8.0635(6) Å, V = 1366.93(17) Å^3^, *Z* = 4, *Z*′ = 1, *R*-factor (%) 7.80, 24.0. The crystal exhibited twinning and aldehyde disorder in one of the aldehyde CHO groups and, therefore, was treated using the CrysAlisPro software and the SHELXL14 refinement programs.

In Figure 2b the major orientation of the disordered aldehyde O3A is shown. Only weak intermolecular interactions were present in the crystal structure.

### 2.4. Syntheses of Polyazomethine Polymers (P-I to P-VI)

All polyazomethines derived from aromatic dialdehydes were synthesized under N_2_ atmosphere by solution polycondensation. A typical procedure is as follows: 

The dialdehydic monomers (1 equivalent) were dissolved in 20 mL of DMF and 10 mL of toluene. *p*-Toluenesulfonic acid (TsOH) monohydrate was added in a catalytic amount in the reaction mixture. After that, the equimolar diamine (1 equivalent) solution in 10 mL DMF was added dropwise with continuous stirring. The reaction was distilled for azeotropic water removal with toluene using a Dean–Stark trapper. After 6 h reflux, TLC was carried out to check the presence of for any residual monomers, using CHCl_3_ eluent. The precipitated product was directly poured onto 300 mL of ice-cold water. It was filtered, washed with methanol, and dried at 40 °C for 6 h. 

On the other hand, if the polymers were soluble in DMF, then upon reaction completion, excess DMF was reduced to half by rotary evaporation followed by precipitation in ice cold H_2_O. Then, the product was filtered, water washed, recrystallized from ethanol/chloroform, and subsequently dried at 40 °C for 6 h.

## 3. Results and Discussion

### 3.1. Syntheses and Characterization of Monomers (3a, 3b, and 3c)

**Figure 3:**
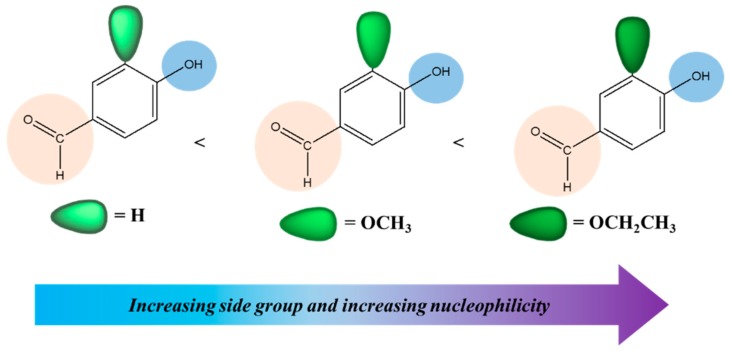
The simplified illustration of increasing side group and nucleophilicity affecting the rate of reaction.

The aromatic bis-aldehyde monomers **3a**, **3b**, and **3c** were conveniently synthesized in the presence of anhydrous K_2_CO_3_ in DMF solvent by reaction of 4-fluorobenzaldehyde (**1**) with 4-hydroxybenzaldehyde (**2a**), 3-methoxy-4-hydroxybenzaldehyde (**2b**), and 3-ethoxy-4-hydroxy-benzaldehyde (**2c**), respectively (Figure 1). During the syntheses of these monomers it was noted that the reaction was completed quickly for **3a** as compared to **3b** and **3c**. Figure 3 shows a simplified illustration of how steric and electronic effects operated for the reaction. Although, going from **2a** through **2c** the nucleophilic character increased because of the electron donating effect of –O*R* groups, which facilitate the concurrent attack on electrophilic carbon of Ar–F, but the simultaneous increase in bulkiness of the groups caused hindrance to F^−^ substitution and, hence, led to a delayed time of reaction.

**Figure 4:**
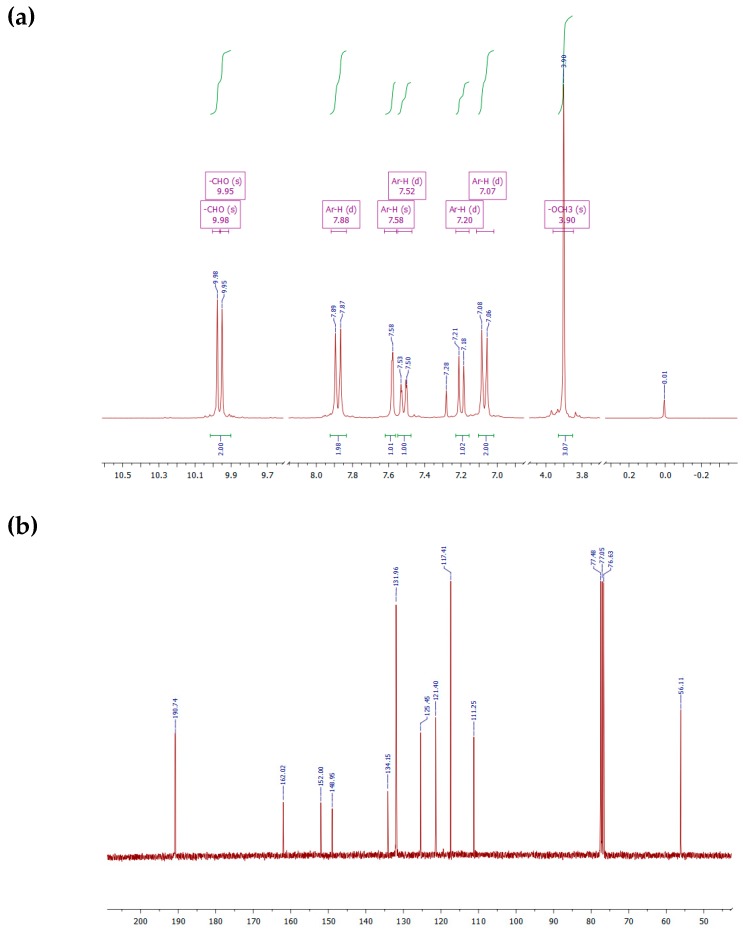
(**a**) ^1^H NMR spectrum of **3b**, and (**b**) ^13^C NMR spectrum of **3b** in CDCl_3._

The FT-IR spectroscopic and elemental analysis data of monomers (**3a-c**) were found to be in accordance with the proposed structures. Figure 4a shows the proton NMR spectrum of monomer **3b**. The resonance lines for protons integrating 2H at δ 9.98 and 9.95 ppm were assigned to the aldehydic protons of two aromatic rings because of their different electronic environment. The two doublets at δ 7.88 and 7.07 ppm each with a coupling constant 8.6 Hz were assigned to the symmetrically substituted benzene ring (see Figure 1 and Figure 4a). The singlet at δ 7.58 ppm, due to 1H, was assigned to H-2 (Figure S1 in Supplementary Materials showing labelling), while the two doublets at δ 7.52 ppm with *J* = 9.7 Hz, and 7.20 ppm with *J* = 8.0 Hz, both integrating 1H were assigned to H-5 and H-6. The CDCl_3_ solvent gave a singlet at δ 7.28 ppm. The singlet appearing at 3.90 ppm, due to 3H, was from the –OCH_3_ protons. In a similar way, the signals of **3c** were assigned as for **3b,** whereas the spectrum of **3a** was simple in that it showed one singlet at δ 9.99 ppm, due to 2H, and two doublets δ 7.94 and 7.19 ppm with couplings constants at 8.0 Hz, thus confirming the symmetrical substituted aromatic rings (Figures S2 and S3, Supplementary Materials). ^13^C NMR spectrum of monomer **3b** is shown in Figure 4b. In this spectrum, the peak at 190.74 ppm was assigned to –CHO carbon atoms, while the rest of the peaks from 162.02–111.25 ppm were ascribed to the aromatic carbons, the triplet at 77 ppm was due to the solvent, CDCl_3_, whereas the single peak at 56.11 ppm was due to the methyl carbon. The assignments of the other two monomers were assigned in the same way (Figures S4 and S5, Supplementary Materials).

### 3.2. Syntheses of Polyazomethines

**Figure 5:**
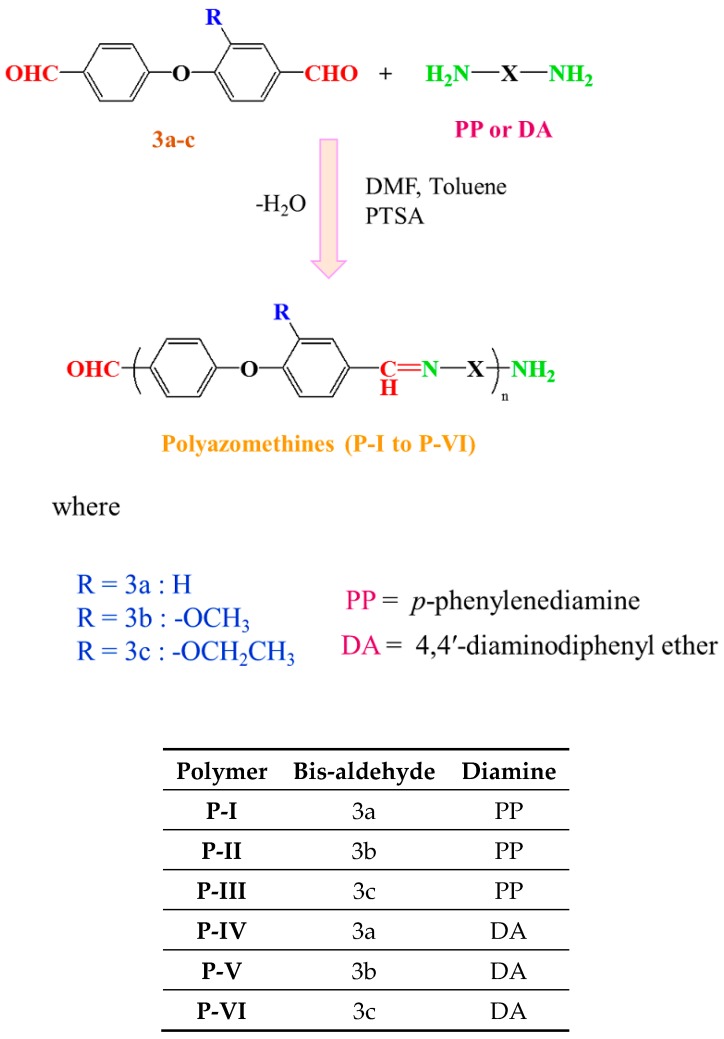
Synthetic scheme of polyazomethines from bis-aldehydes and diamines.

**Figure 6:**
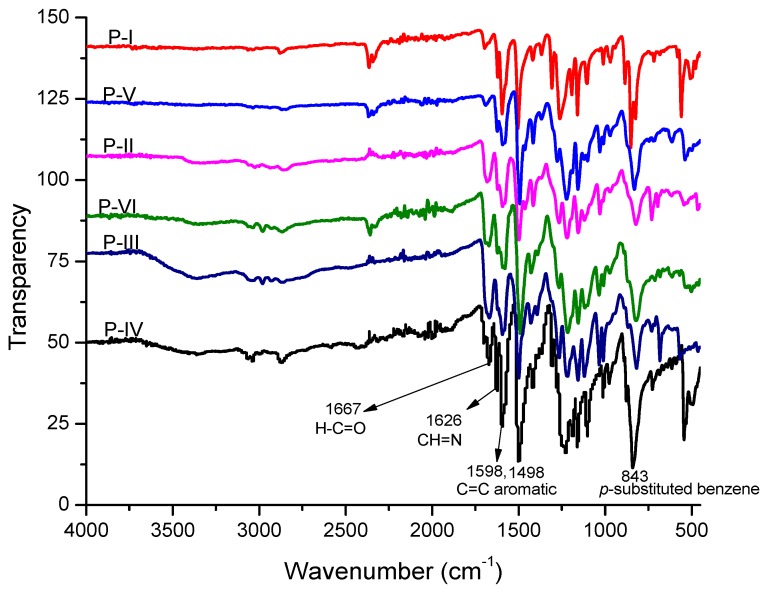
FT-IR spectra of polyazomethines. The spectra are shifted along the direction of intensity for clarity.

All polyazomethines (**P-I** to **P-VI**) were synthesized by solution phase polycondensation under N_2_ atmosphere. Bis-aldehyde monomer (0.1 mmol) was dissolved in 20 mL of dried DMF and 10 mL of dried toluene. Subsequently, *p*-toluenesulfonic acid monohydrate (PTSA) was added in catalytic amount (20 mg). Then, the diamine (0.1 mmol) solution in 10 mL of DMF was added dropwise with continuous stirring. The reaction was gently heated under reflux conditions for 6 h. Then, the product was directly precipitated by pouring it onto 300 mL of ice-cold water. Afterward, the product was filtered, washed with MeOH, and dried at 40 °C for 6 h before further characterization. The synthetic scheme of polyazomethines is shown in Figure 5. The solubility and viscosity data of these polyazomethines are presented in Tables S1 and S2 (Supplementary Materials). The FT-IR spectra of polymers are presented in Figure 6, and the values of vibrational frequencies are listed below along with ^1^H NMR data.

**P-I:** FT-IR (KBr): υ(end group HC=O str.), 1691 cm^−1^; *υ*(C=N str.), 1620 cm^−1^; *υ*(aromatic C=C), 1591, 1503 cm^−1^; *υ*(C–O str.), 1257 cm^−1^; *υ*(aromatic ring), 855 cm^−1^. ^1^H NMR (300 MHz, D_2_SO_4_, *δ* ppm): 8.24 (s, azomethine), 7.93–6.70 (m, aromatic protons).

**P-II:** FT-IT (KBr): *υ*(end group HC=O), 1684 cm^−1^; *υ*(C=N str.), 1606 cm^−1^; *υ*(aromatic C=C), 1584, 1491 cm^−1^; *υ*(C–O str.), 1221, 1150 cm^−1^; *υ*(aromatic ring), 823 cm^−1^. ^1^H NMR (300 MHz, D_2_SO_4_, δ ppm): 8.31 (s, azomethine), 7.61–6.67 (m, aromatic protons).

**P-III:** FT-IT (KBr): *υ*(merged peak of HC=O and C=N str.), 1691–1669 cm^−1^; *υ*(aromatic C=C), 1591, 1499 cm^−1^; *υ*(C–O str.), 1214, 1157, 1122 cm^−1^; *υ*(aromatic ring), 816 cm^−1^. ^1^H NMR (300 MHz, D_2_SO_4_, δ ppm): 8.43 (s, azomethine), 7.62–6.73 (m, aromatic protons).

**P-IV:** FT-IT (KBr): *υ*(end group HC=O), 1684 cm^−1^; *υ*(C=N str.), 1626 cm^−1^; *υ*(aromatic C=C), 1585, 1506 cm^−1^; *υ*(C–O str.), 1214 cm^−1^; *υ*(aromatic ring), 822 cm^−1^. ^1^H NMR (300 MHz, D_2_SO_4_, δ ppm): 8.32 (s, azomethine), 7.92–7.07 (m, aromatic protons).

**P-V:** FT-IT (KBr): *υ*(end group HC=O), 1691 cm^−1^; *υ*(C=N str.), 1620 cm^−1^; *υ*(aromatic C=C), 1581, 1491 cm^−1^; *υ*(C–O str.), 1229, 1150 cm^−1^; *υ*(aromatic ring), 829 cm^−1^. ^1^H NMR (300 MHz, D_2_SO_4_, δ ppm): 8.33 (s, azomethine), 7.60–6.49 (m, aromatic protons), 3.54 (s, –OCH_3_).

**P-VI:** FT-IT (KBr): *υ*(merged peak of HC=O and C=N str.), 1699–1676 cm^−1^; *υ*(aromatic C=C), 1584, 1498 cm^−1^; *υ*(C–O str.), 1221, 1150 cm^−1^; *υ*(aromatic ring), 843 cm^−1^. ^1^H NMR (300 MHz, D_2_SO_4_, δ ppm): 8.25 (s, azomethine), 7.97–6.71 (m, aromatic protons).

The disappearance and/or only weak absorption peaks of the carbonyl group of aldehydes confirmed the synthesis of polymers. The weak absorptions were due to the carbonyl end groups on the macrochains. This infers that the aldehydic carbonyl groups were utilized in the successful formation of azomethine linkages. On the other hand, the presence of medium intensity absorption peaks in the range of υ (1620–1627 cm^−1^) were ascribed to –CH=N– bonds of azomethine. Similarly, the absence of –NH_2_ absorption peaks also had additive effects to the aforesaid formation of polymers. Introduction of ethoxy side groups into the polyimine structure shifted the azomethine peak toward higher wavenumbers, as indicated by the broad merged peaks for **P-III** and **P-VI**, which showed that the end group HC=O and CH=N bond stretching frequencies overlapped, whereas the methoxy side groups did not affect the imine peaks in comparison to the structure containing no side groups.

**Figure 7:**
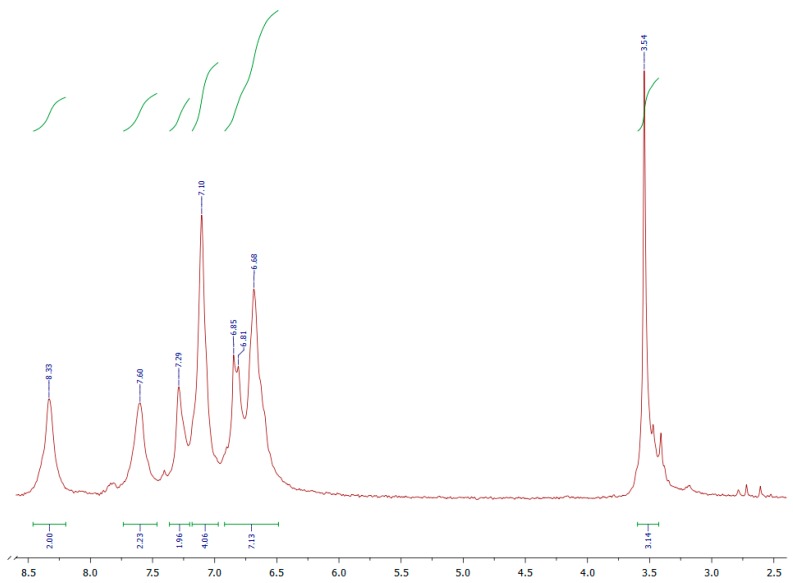
^1^H NMR spectrum of polyazomethine polymer **P-V** in CDCl_3_.

The presence of an imine bond was also confirmed by ^1^H NMR spectroscopy. The positions of peaks characteristic of an imine bond varied in the spectral range of *δ* 8.43–8.24 ppm. Figure 7 shows the proton NMR spectrum of polyazomethine P-V. The spectrum clearly reveals a singlet at δ 8.33 ppm associated with an azomethine group (–CH=N–), whereas the aromatic protons gave signals at δ 7.60–6.49 ppm. The singlet arising at δ 3.54 ppm was clearly assignable to the methoxy group in the continuous chain of wholly aromatic rings. The ^1^H NMR spectrum confirmed the formation of the imine bond. For the rest of the polymers, the imine protons were found at δ 8.43–8.24 ppm, whereas the aromatic protons signals overlapped, and we were not able to separate them.

### 3.3. Powder X-ray Diffraction

**Figure 8:**
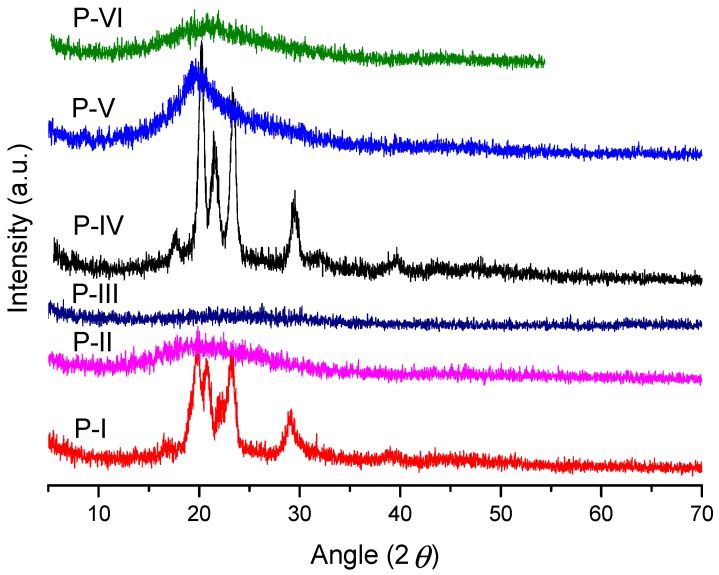
Powder XRD of polyazomethine-synthesized aromatic bis-aldehyde monomers **3a**, **3b**, and **3c**.

Powdered X-ray diffraction is the most important technique to determine the degree of crystallinity in polymers. The intensities, peak positions, shapes, and widths give important information about the structure of the polymeric material. The X-ray diffraction profiles of powdered polyazomethines samples were conducted at room temperature to get information about the semicrystallinity of polyazomethines. The X-ray diffraction patterns of **P-I** and **P-IV** showed three peaks at 2θ = 17.67–23.40° and a fourth peak at 29.40°, as observed in Figure 8**.** Wide-angle X-ray diffraction patterns allowed to conclude that the investigated polyazomethines **P-I**, **P-IV,** and **P-V** had semicrystalline structures, as expected, and this result was consistent with previous reports [18,42]. The polymer synthesized from **3a** monomer (viz **P-I** and **P-IV**) showed the highest crystallinity, those synthesized from **3b** (i.e., **P-II** and **P-V**) showed little or no crystallinity, whereas those synthesized from **3c** (i.e., **P-III** and **P-VI**) showed a totally amorphous nature. This clearly shows the role of symmetry of the polymeric macrochain in determining the semicrystallinity of the polymer. The polymers of **3b** and **3c** having –OCH_3_ and –OC_2_H_5_ side groups to the main chain hindered their close packing and, thus, made them amorphous.

### 3.4. UV–Vis and Photoluminescence Measurements

**Figure 9:**
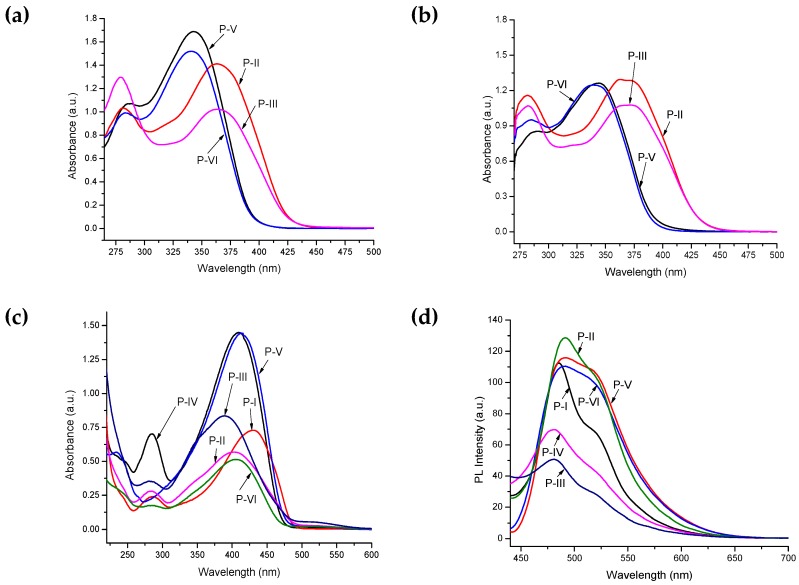
UV–vis spectra of dilute solutions of polyazomethines in (**a**) chloroform, (**b**) DMF, (**c**) sulfuric acid, and (**d**) photoluminescence spectra of polyazomethines using 420 nm as excitation wavelength.

The UV–vis spectra of the polyazomethines were recorded at very dilute solutions. They produced maximum absorption at 285–365 nm in CHCl_3_ (Figure 9a) and at 282–373 nm in DMF (Figure 9b). The solutions of partially soluble azomethines were filtered before recoding absorption spectra. The polymers synthesized from the **3a** monomer showed complete insolubility in both chloroform and DMF. It is evident from Figure 9a,b and Table 1 that the value of electronic absorption bands of polyazomethines depended on the polarity of the solvent. Therefore, absorption bands measured in DMF revealed an overall red shift (also known as positive solvatochromism), which is believed to be due to the strong interactions between the polar sites of the solvent and the highest occupied molecular orbital (HOMO) and lowest unoccupied molecular orbital (LUMO) levels of polymers. Acidochromic behavior was found in the UV–visible spectra of macromolecular azomethines in sulfuric acid (Figure 9c). The drift in electronic absorption maxima of these azomethines to bathochromic region suggests the extension of delocalized π-conjugation length. Electronic spectra recorded in sulfuric acid revealed that the charge transfer from Bronsted acid to a lone pair of electrons on a nitrogen in azomethine occurred. It is suggested that protonation of the azomethine linkage resulted in a change of the planarity of the polymer backbone and, thus, led to increased π-electronic cloud delocalization over a wider range, which caused a marked bathochromic shift.

Photoluminescence (PL) measurements (Figure 9d) were performed using 420 nm as the excitation wavelength, and emission spectra were measured from 440 nm onward. All the azomethines revealed PL features and emitted blue light in the range of 481–492 nm. The highest photoluminescence intensity was found for **P-II,** while lowest was observed for **P-III**. The difference in energies of the absorption maxima and emission maxima (known as Stokes shift) were shown in terms of wavelength and occurred in the range from 54 to 92 nm, indicating an energy decline during electronic transitions. This energy loss can be due to the excimer formation as demonstrated by the bathochromic effect and enlargement of the emission bands (see Figure 9d).

### 3.5. Thermal Properties

The thermal stability of the polyazomethines was investigated by thermogravimetric analysis (TGA), and the data are tabulated in Table 2. Thermal data displayed that the polymers were remarkably thermally stable. The thermal stability of these polymers is shown in Table 2 in terms of *T*_on_, *T*_20%_, *T*_max_, and *T*_F_ in degrees, and percent char yield showing onset of degradation, 20% weight loss, maximum degradation temperature, and final degradation temperatures, respectively. The onset temperature of degradation (*T*_on_) for these polymers was in the range from 150 to 450 °C. The maximum stability to initial heating was shown by polymers synthesized from monomer **3a** (i.e., **P-I** and **P-IV**) viz 300 and 450 °C and minimum stability showed by polymers synthesized from **3c** having the –OC_2_H_5_ side group. The maximum degradation temperature (*T*_max_) for polyazomethines was between the range of 475 and 650 °C. The high thermal stability of these polymers can be ascribed to the rigidity of the monomers and effective staking of aromatic moieties in macrochains.

### 3.6. Electrical Conductivity

The electrical conductivity measurements of the synthesized pristine polyazomethines were measured in air atmosphere in solid state on a Keithley 2400 instrument using pelletized powdered samples. The pellets of the polymer samples were prepared by pressing the powdered sample for 5 min at 4.9 metric ton pressure using a hydraulic press. Electrical connections on pellets were made with Cu wires, which were abraded with fine grit silicon carbide sandpaper, and placed equidistant from each other. The Cu connections on pellets were fixed with a conducting paste of silver. The current (ranging 1–3 µA) was forced through the two points of the probe, labelled as 1 and 4, and measured the voltage (V) as the output signal through points 2 and 3 [43]. Subsequently, the conductance and conductivities of polymer samples were calculated from the slopes of current versus voltage (I-V) curves, which are shown in Figure S6 (Supplementary Materials). The values of resistance, conductance, and conductivity are tabulated in Table 3. The pellet dimensions were taken as: diameter = 1.25 cm, radius (*r*) = 0.625 cm, area (*A*) = 1.22718 cm^2^, and distance between two probes (*L*) = 0.3 cm.

The electrical conductivities of the undoped polyimines have been reported to be 10^−11^ Scm^−1^, while those of the doped samples with iodine were 10^−6^ Scm^−1^ [6,8,44]. However, we have found the electrical conductivities of these pelletized polyazomethine in the range from 4.0 × 10^−5^ to 6.4 × 10^−5^ Scm^−1^, which is ten times higher than the conductivities of any pristine polyazomethines reported so far. Although the effect of doping on the electric properties of polyazomethines have been presented in numerous papers, according to our best knowledge, no one has reported the conductivity of undoped polyazomethines as high as 6.4 × 10^−5^ Scm^−1^. The low conductivity values were attributed to the low degree of conjugation in the polymers caused by nonplanarity of the polymer chains. But in actual cases, the nonplanarity of the CH=N bond is not the cause of low conductivity, as was demonstrated by our synthesized polymers—they showed conductivity in the 10^−5^ Scm^−1^ range. Rather, the low conductivity was caused by different unconjugated aliphatic spacers. And when the polymers were doped with iodine in the doping process, the electron emitting imine nitrogen and the electron pulling iodine coordinated to form a radical cation (polaron) structure. The electron vacancy formed because this polaron facilitates the electron transfer from one chain to another, thus causing an increase in the electrical conductivity. Still, the maximum conductivity of iodine-doped polymers is reported to be 10^−6^ Scm^−1^ [32,34,44,45]. This value is comparable to the poly(azomethine-ester)s [46], which suggest that the ester linkage is nonconducting, just as aliphatic spacers. So, we present wholly aromatic polyazomethines—without any aliphatic spacer—whose conductivity in the pristine form is ten-fold greater than the iodine-doped polyazomethines. The polymer **P-III** has the highest conductivity value of 6.4 × 10^−5^ Scm^−1^, which probably is due the straight pathway of electronic conduction provided by azomethine linkages between **3c** and **PP** monomers. In general, the electrical conductivities of the polymers synthesized from the **PP** monomer were higher than those synthesized from **DA**. The lower values of conductivities of polymers of **DA** may be ascribed to the presence of an additional ether linkage, which is less conductive as compared to the azomethine group. Overall, the conductivities of all the polymers were comparable to each other, which indicate the same electronic environment in all these polymers.

## 4. Conclusions

In this paper, we have presented three monomers (along with single crystals of two monomers) and six wholly aromatic polyazomethines, which were tested in pristine form for their electrical conductivity. Methods of synthesis and spectroscopic characterizations of monomers and polyazomethines have been discussed. Synthesis of each polymer was conducted via polycondensation in solution form. The synthesized polymers were orange-yellow or dark brown-yellow powders. Obtained polymers were investigated by ^1^H NMR, FT-IR, UV–vis spectroscopy, and photoluminescence. The results suggest that although the solubility of polymers might slightly hinder their applications, they showed good optical properties. The blue light emitting polymers could be structurally engineered to use in optoelectronics. The polyazomethines exhibited lower band gaps, good emission properties, and high strokes shift values, so they can be used in the preparation of fluorescence sensor material. Wide-angle X-ray diffraction showed their semicrystallinity, while thermal analysis showed good thermal resistance of all polyazomethines. High percent char residue in thermogravimetric analysis is suggestive that the synthesized polyimines have an excellent thermal stability and can be used as potential candidates for high-temperature applications. It was observed that the conductivities of polyazomethines in the solid state were from 4.0 × 10^−5^ to 6.4 × 10^−5^. The semiconducting polyazomethines might be used in electronic, optoelectronic, and photovoltaic applications. We believe that these conjugated polyazomethines will be key functional materials for future applications. 

## Figures and Tables

**Table 1:** Electronic absorption and photoluminescence data of polyazomethine polymers.

Polymer	*λ*_max_ (nm) ^a^	*λ*_max_ (nm) ^b^	*λ*_max_ (nm) ^c^	*λ*_onset_ (nm) ^c^	*E*_g_ (eV) ^c,d^	PL *λ*_max_ (nm) ^c^	Stoke’s Shift (nm)
**P-I**	–	–	432, 286	488	2.54	486	54
**P-II**	365, 281	372, 282	404, 283	487	2.55	491	87
**P-III**	365, 279	373, 282	391, 283	485	2.56	482	91
**P-IV**	–	–	411, 285	477	2.60	481	70
**P-V**	344, 285	345, 287	415	480	2.59	492	77
**P-VI**	341, 282	343, 284	408, 285	471	2.64	492	84

^a^ The absorbance measured in chloroform; ^b^ The absorbance measured in DMF; ^c^ The absorbance measured in sulfuric acid; ^d^ Calculated from the equation: *E_g_* = 1242/*λ_onset_*.

**Table 2:** Thermal degradation data of polyazomethines performed by thermogravimetric analysis.

Polymer	*T*_on_ (°C)	*T*_20%_ (°C)	*T_max_* (°C)	*T*_F_ (°C)	%Char Residue
**P-I**	300	525	525	650	53.3
**P-II**	300	490	650	950	38.5
**P-III**	150	510	550	950	32
**P-IV**	450	520	530	650	46.6
**P-V**	360	430	475	630	57
**P-VI**	125	450	575	600	60

**Table 3:** Calculated resistance, conductance, and conductivity from the current vs voltage (I-V) curves of the polyazomethines polymers.

Polymer	Resistance (R) × 10^3^ Ω	Conductance (G) × 10^−3^ Ohm^−1^	Conductivity (σ) Scm^−1^
**P-I**	4.74437 ± 1.07	0.210	5.1 × 10^−5^
**P-II**	6.08604 ± 1.49	0.164	4.0 × 10^−5^
**P-III**	3.83071 ± 0.81	0.261	6.4 × 10^−5^
**P-IV**	5.12437 ± 0.69	0.195	4.8 × 10^−5^
**P-V**	4.91478 ± 0.82	0.203	5.0 × 10^−5^
**P-VI**	5.28489 ± 0.63	0.198	4.8 × 10^−5^

## Supplementary Materials

The following are available online at https://www.mdpi.com/2073-4360/11/9/1498/s1, Figure S1: Labelling of monomers, Figure S2: Proton NMR of 3a monomer, Figure S3: Proton NMR of 3c monomer, Figure S4: Carbon-13 NMR of 3a monomer, Figure S5: Carbon-13 NMR of 3c monomer, Figure S6: current vs voltage (I-V) curves of polyazomethines polymers, Table S1: Organosolubility of polyazomethines in common organic solvents, Table S2: Viscometric Data of polyazomethine Polymers, The single crystal data for the two isomers are provided with CCDC reference codes 1509984 and 1509985. Copies available, e-mail: deposit@ccdc.cam.ac.uk.
