# Supplementary material for: Synthesis, Crystal Structures, and Spectroscopic Characterization of Bis-aldehyde Monomers and Their Electrically Conductive Pristine Polyazomethines
Source: Polymers (Basel). 2019 Sep 13;11(9):1498. doi: 10.3390/polym11091498 (PMC6780126; doi:10.3390/polym11091498)
Supplement: Supplementary file 1 [file polymers-11-01498-s001.pdf]

polymers

Supporting Information

Synthesis, Crystal Structures and Spectroscopic Characterization of *bis*-aldehyde Monomers and Their Electrically Conductive Pristine Polyazomethines

Abdul Hafeez[a], Zareen Akhter[a*], John F. Gallagher[b], Nawazish Ali Khan[c], Asghari Gul[d] and Faiz Ullah Shah[e*]

[a.] Department of Chemistry, Quaid-i-Azam University, Islamabad 45320, Pakistan
[b.] School of Chemical Sciences, Dublin City University, Glasnevin, Dublin 9, Ireland
[c.] Materials Science Laboratory, Department of Physics, Quaid-i-Azam University, Islamabad 45320, Pakistan
[d.] Department of Chemistry, COMSATS University, Islamabad, Pakistan.
[e.] Chemistry of Interfaces, Luleå University of Technology, 971 87 Luleå, Sweden

[*.] Corresponding authors. zareenakhter@yahoo.com and faiz.ullah@ltu.se

3a

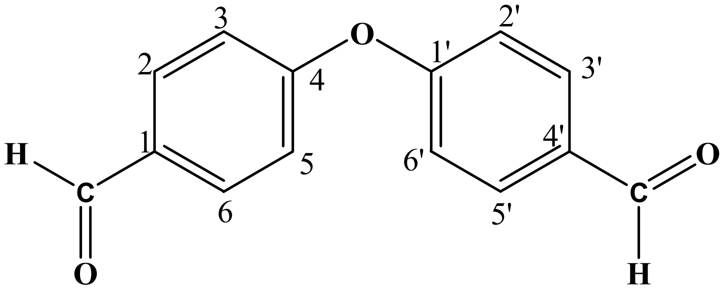

3b

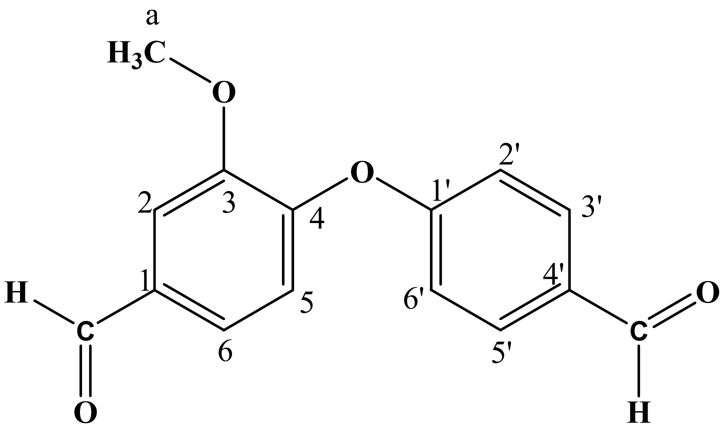

3c

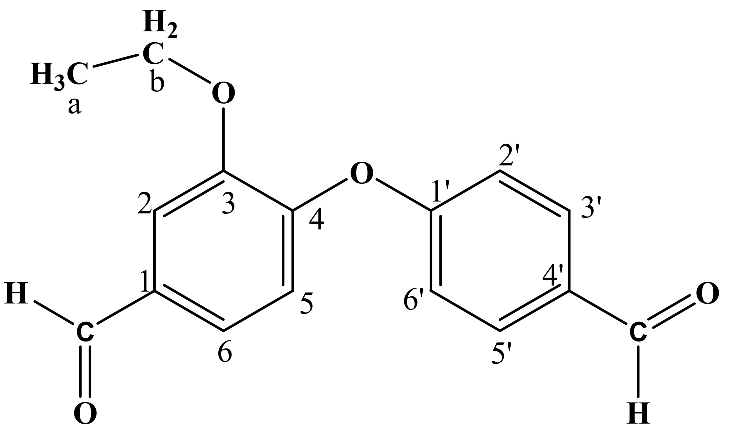

Figure S1. Labelling of monomers.

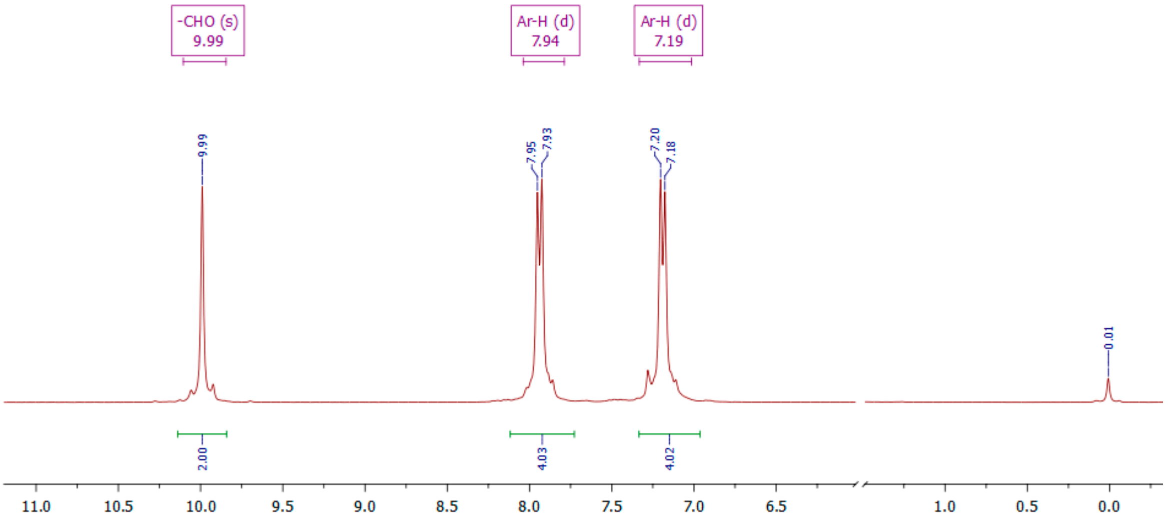

Figure S2. Proton NMR of 3a monomer.

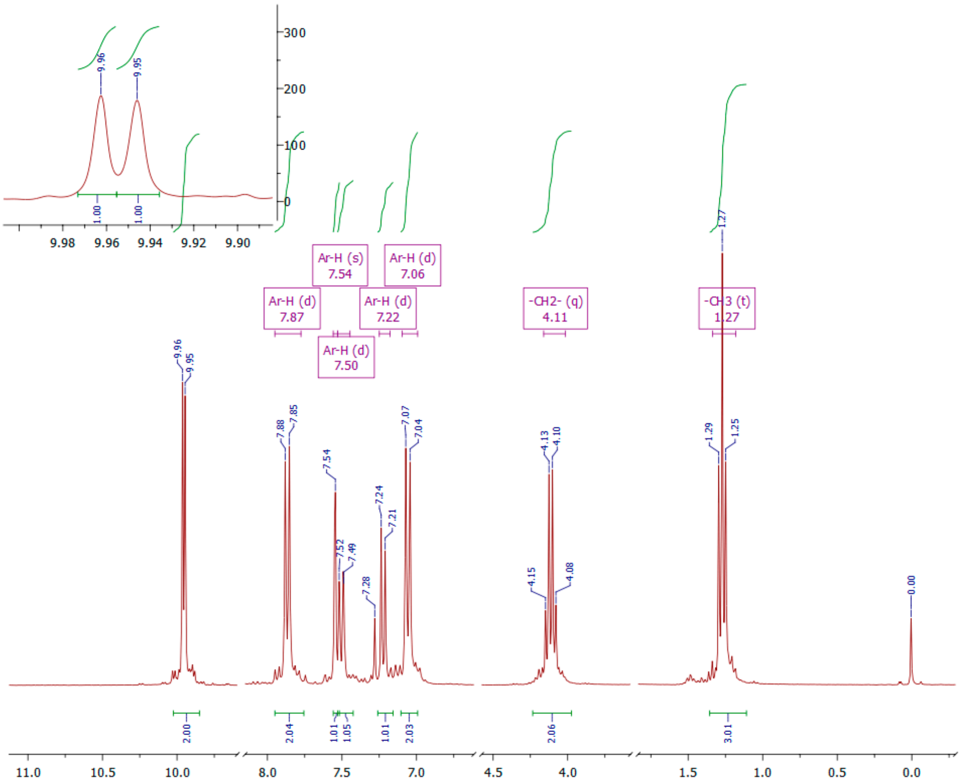

Figure S3. Proton NMR of 3c monomer.

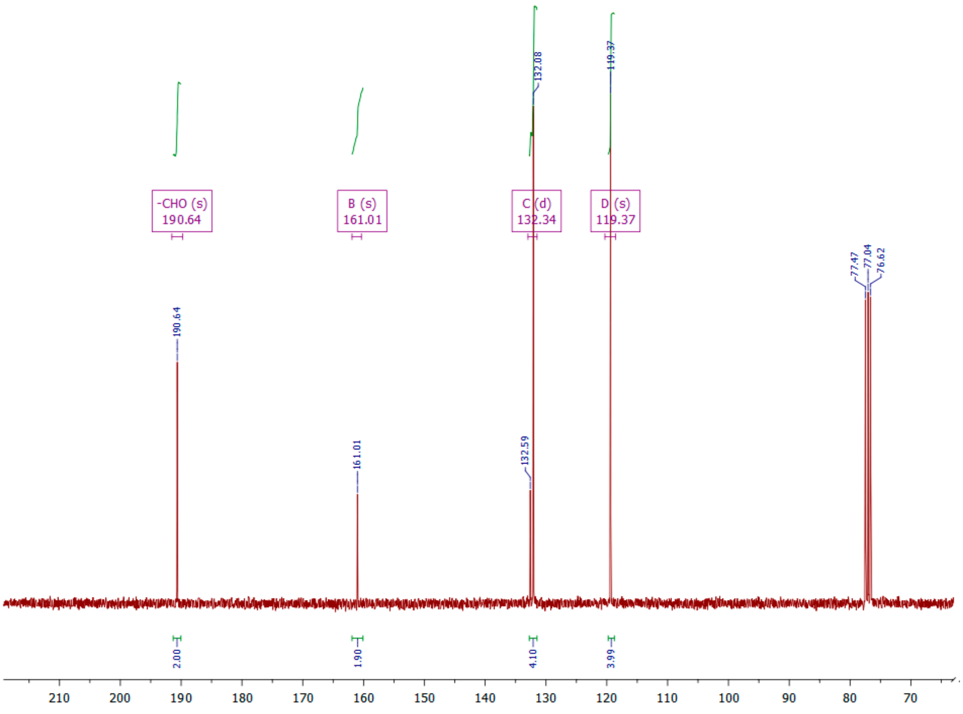

Figure S4. Carbon-13 NMR of 3a monomer.

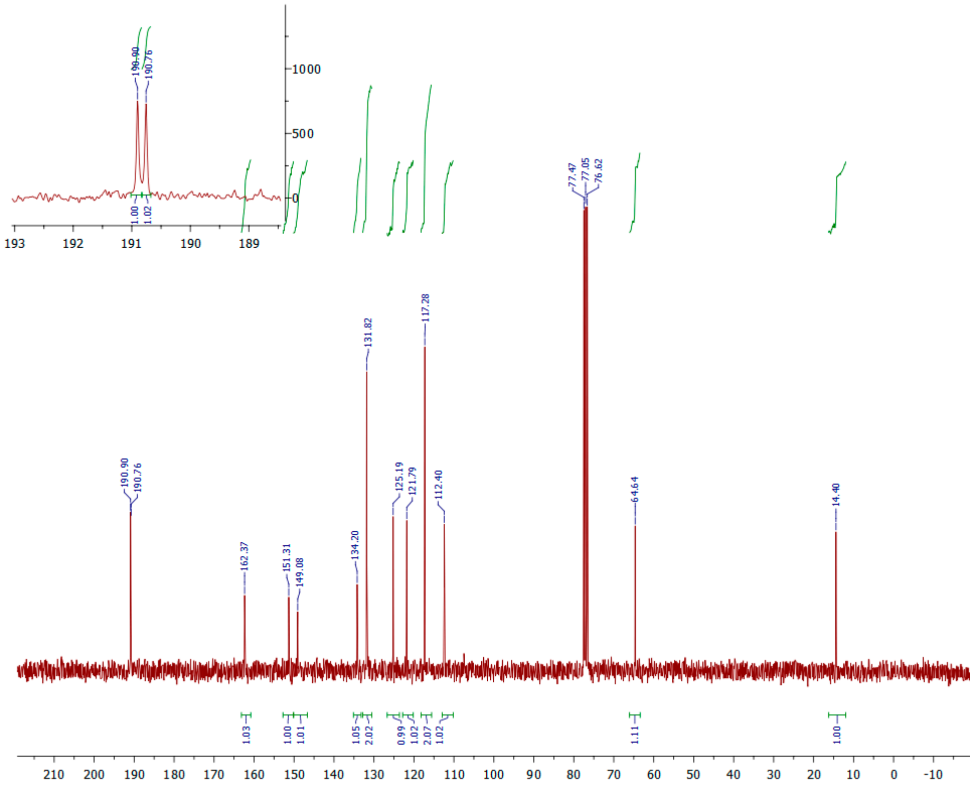

Figure S5. Carbon-13 NMR of 3c monomer.

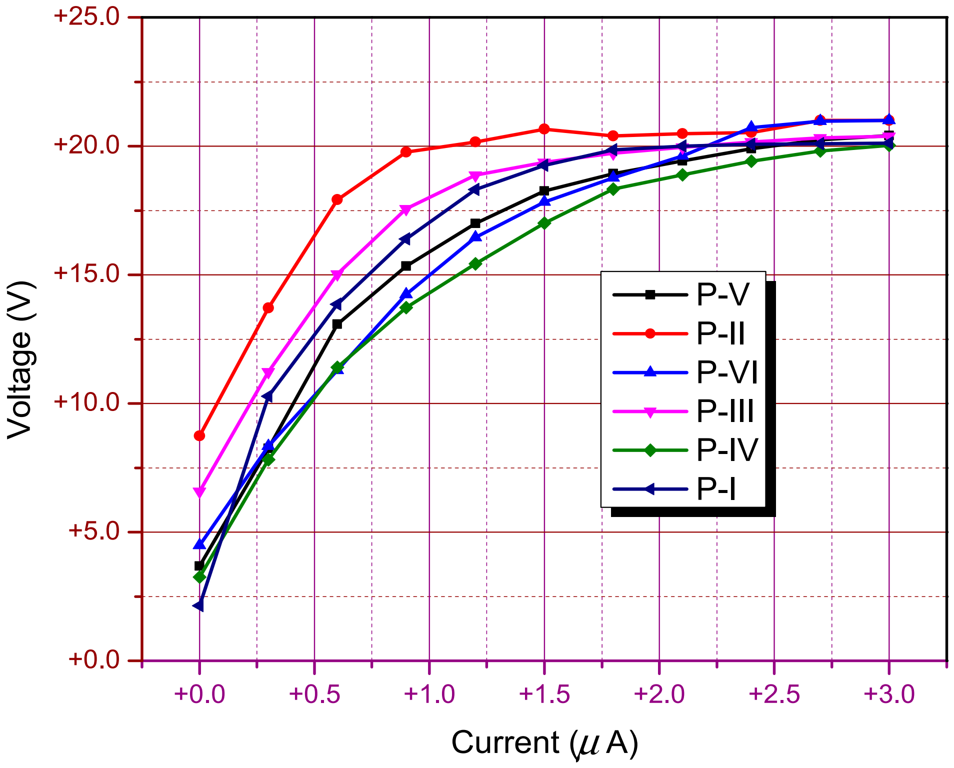

Figure S6. current vs voltage (I-V) curves of polyazomethines polymers.

Organosolubility and inherent viscosities

The organosolubility of polymeric azomethines was checked at 30 °C in various solvents such as *n*-hexane, DMF, DMSO, NMP, $CHCl_3$ and sulfuric acid by dissolving 10 mg powdered polymer sample in 1 mL of solvent and the results are presented in Table S1. It was found that the polymers synthesized from non-substituted aromatic dialdehyde (*i.e.* 3a) were almost insoluble in most of the solvents whereas others having $–OCH_3$, and $–OC_2H_5$ showed reasonable solubility in all the solvents. The limited solubility of wholly aromatic polyazomethines may be attributed to the chain rigidity, regularity and inter-chain packing efficiency. It may also be noted that the polymer **P-I** and **P-IV** showed semi-crystalline characteristics in powder XRD which reinforces the fact that polymer chains stacked over one another. However, all the polymeric azomethines showed good solubility in *conc.* H_2SO_4 at room temperature and Table S1 shows the respective solubility behavior of polyazomethines in aforementioned solvents. The polymers had viscosity in the range 1.98-1.72, as shown in Table S2.

Table S1. Organosolubility of polyazomethines in common organic solvents.

Polymer	n-hexane	DMF	DMSO	NMP	$CHCl_3$	H_2SO_4
**P-I**	– – h	– – h	– – h	– – h	– – h	+ + +
**P-II**	– – h	+ + +	+ + h	+ + h	+ + +	+ + +
**P-III**	– – h	+ + +	+ + +	+ + +	+ + +	+ + +
**P-IV**	– – h	– – h	– – h	– – h	– – h	+ + +
**P-V**	– – h	+ – h	+ – h	+ – h	+ – h	+ + +
**P-VI**	– – h	+ + h	+ + h	+ – h	+ + +	+ + +

+ + + = soluble at room temperature, + – h = partially soluble on heating
– – h = insoluble on heating, + + h = soluble on heating

Table S2. Viscometric Data of polyazomethine Polymers.

Code	η_{rel}	η_{sp}	η_{red} (dl/g)	η_{inh} (dl/g)
**P-I**	1.17	0.17	0.86	1.77
**P-II**	1.25	0.25	1.28	1.84
**P-III**	1.12	0.12	0.60	1.72
**P-IV**	1.136	0.14	0.68	1.74
**P-V**	1.45	0.45	2.25	1.98
**P-VI**	1.18	0.18	0.94	1.78
